# Supplementary material for: Modeling glioblastoma heterogeneity as a dynamic network of cell states
Source: Mol Syst Biol. 2021 Sep 16;17(9):e10105. doi: 10.15252/msb.202010105 (PMC8444284; doi:10.15252/msb.202010105)
Supplement: Supplementary file 6 — Source Data for Figure 5 [file MSB-17-e10105-s004.zip › Figure5A_sourcedata/GSEA_3071/hallmarks_stateII.GseaPreranked.1623742536692/HALLMARK_ESTROGEN_RESPONSE_EARLY.html]

Details for gene set HALLMARK\_ESTROGEN\_RESPONSE\_EARLY[GSEA]

|  || Dataset | state23071 |
| Phenotype | NoPhenotypeAvailable |
| Upregulated in class | na\_pos |
| GeneSet | HALLMARK\_ESTROGEN\_RESPONSE\_EARLY |
| Enrichment Score (ES) | 0.22642341 |
| Normalized Enrichment Score (NES) | 1.2325295 |
| Nominal p-value | 0.17061612 |
| FDR q-value | 0.26477572 |
| FWER p-Value | 0.976 |
Table: GSEA Results Summary

  

Fig 1: Enrichment plot: HALLMARK\_ESTROGEN\_RESPONSE\_EARLY      
 Profile of the Running ES Score & Positions of GeneSet Members on the Rank Ordered List

  

| PROBE | GENE SYMBOL | GENE\_TITLE | RANK IN GENE LIST | RANK METRIC SCORE | RUNNING ES | CORE ENRICHMENT || 1 | RBBP8 |  |  | 86 | 1.100 | 0.0093 | Yes |
| 2 | MYBL1 |  |  | 102 | 1.043 | 0.0346 | Yes |
| 3 | DLC1 |  |  | 208 | 0.868 | 0.0327 | Yes |
| 4 | TFF3 |  |  | 225 | 0.851 | 0.0524 | Yes |
| 5 | FKBP5 |  |  | 375 | 0.695 | 0.0348 | Yes |
| 6 | UGCG |  |  | 388 | 0.685 | 0.0508 | Yes |
| 7 | IGFBP4 |  |  | 401 | 0.678 | 0.0667 | Yes |
| 8 | PMAIP1 |  |  | 426 | 0.666 | 0.0793 | Yes |
| 9 | NXT1 |  |  | 438 | 0.657 | 0.0949 | Yes |
| 10 | OLFML3 |  |  | 439 | 0.657 | 0.1131 | Yes |
| 11 | CALB2 |  |  | 485 | 0.636 | 0.1197 | Yes |
| 12 | FLNB |  |  | 488 | 0.633 | 0.1368 | Yes |
| 13 | SLC9A3R1 |  |  | 497 | 0.630 | 0.1523 | Yes |
| 14 | PAPSS2 |  |  | 529 | 0.612 | 0.1617 | Yes |
| 15 | FHL2 |  |  | 541 | 0.607 | 0.1758 | Yes |
| 16 | PPIF |  |  | 639 | 0.560 | 0.1673 | Yes |
| 17 | MICB |  |  | 655 | 0.550 | 0.1789 | Yes |
| 18 | PRSS23 |  |  | 666 | 0.546 | 0.1916 | Yes |
| 19 | MYOF |  |  | 730 | 0.523 | 0.1906 | Yes |
| 20 | SLC2A1 |  |  | 787 | 0.503 | 0.1907 | Yes |
| 21 | MYC |  |  | 790 | 0.502 | 0.2041 | Yes |
| 22 | FKBP4 |  |  | 806 | 0.495 | 0.2142 | Yes |
| 23 | BCL2 |  |  | 813 | 0.494 | 0.2264 | Yes |
| 24 | ISG20L2 |  |  | 1035 | 0.432 | 0.1836 | No |
| 25 | AKAP1 |  |  | 1091 | 0.419 | 0.1817 | No |
| 26 | PODXL |  |  | 1155 | 0.406 | 0.1773 | No |
| 27 | FOXC1 |  |  | 1271 | 0.383 | 0.1595 | No |
| 28 | MYBBP1A |  |  | 1371 | 0.362 | 0.1450 | No |
| 29 | WWC1 |  |  | 1384 | 0.360 | 0.1520 | No |
| 30 | SLC7A5 |  |  | 1434 | 0.351 | 0.1497 | No |
| 31 | KLF10 |  |  | 1662 | 0.315 | 0.1022 | No |
| 32 | CCND1 |  |  | 1753 | 0.304 | 0.0883 | No |
| 33 | SLC7A2 |  |  | 1880 | 0.288 | 0.0651 | No |
| 34 | TPBG |  |  | 1914 | 0.284 | 0.0648 | No |
| 35 | RRP12 |  |  | 1933 | 0.281 | 0.0682 | No |
| 36 | XBP1 |  |  | 1995 | 0.273 | 0.0606 | No |
| 37 | GFRA1 |  |  | 2303 | -0.259 | -0.0083 | No |
| 38 | SYNGR1 |  |  | 2325 | -0.262 | -0.0062 | No |
| 39 | SH3BP5 |  |  | 2336 | -0.263 | -0.0014 | No |
| 40 | CXCL12 |  |  | 2350 | -0.264 | 0.0028 | No |
| 41 | GJA1 |  |  | 2366 | -0.266 | 0.0065 | No |
| 42 | TOB1 |  |  | 2580 | -0.298 | -0.0381 | No |
| 43 | IGF1R |  |  | 2700 | -0.316 | -0.0588 | No |
| 44 | KDM4B |  |  | 2976 | -0.369 | -0.1167 | No |
| 45 | FARP1 |  |  | 3001 | -0.373 | -0.1122 | No |
| 46 | ABAT |  |  | 3075 | -0.391 | -0.1194 | No |
| 47 | NAV2 |  |  | 3083 | -0.393 | -0.1102 | No |
| 48 | GREB1 |  |  | 3177 | -0.422 | -0.1216 | No |
| 49 | AR |  |  | 3204 | -0.429 | -0.1161 | No |
| 50 | SLC26A2 |  |  | 3212 | -0.431 | -0.1058 | No |
| 51 | RPS6KA2 |  |  | 3264 | -0.444 | -0.1061 | No |
| 52 | ADCY1 |  |  | 3299 | -0.457 | -0.1018 | No |
| 53 | SVIL |  |  | 3490 | -0.537 | -0.1340 | No |
| 54 | SLC39A6 |  |  | 3502 | -0.543 | -0.1216 | No |
| 55 | OLFM1 |  |  | 3521 | -0.554 | -0.1106 | No |
| 56 | CELSR2 |  |  | 3550 | -0.570 | -0.1017 | No |
| 57 | RETREG1 |  |  | 3555 | -0.573 | -0.0868 | No |
| 58 | BCL11B |  |  | 3656 | -0.651 | -0.0935 | No |
| 59 | MPPED2 |  |  | 3701 | -0.685 | -0.0853 | No |
| 60 | RAB31 |  |  | 3757 | -0.737 | -0.0785 | No |
| 61 | RHOBTB3 |  |  | 3838 | -0.829 | -0.0752 | No |
| 62 | FOS |  |  | 3911 | -0.966 | -0.0662 | No |
| 63 | CHPT1 |  |  | 3923 | -1.006 | -0.0409 | No |
| 64 | NBL1 |  |  | 3992 | -1.204 | -0.0243 | No |
| 65 | TUBB2B |  |  | 4063 | -1.810 | 0.0087 | No |
Table: GSEA details [plain text format]

  

Fig 2: HALLMARK\_ESTROGEN\_RESPONSE\_EARLY: Random ES distribution      
 Gene set null distribution of ES for **HALLMARK\_ESTROGEN\_RESPONSE\_EARLY**
